# Magnetization Transfer Ratio in the Typically Developing Pediatric Spinal Cord: Normative Data and Age Correlation

**DOI:** 10.1111/jon.70019

**Published:** 2025-02-09

**Authors:** Sara Naghizadeh Kashani, Iswarya Vel, Zahra Sadeghi Adl, Shiva Shahrampour, Devon Middleton, Mahdi Alizadeh, Laura Krisa, Scott Faro, Slimane Tounekti, Julien Cohen‐Adad, Feroze B. Mohamed

**Affiliations:** ^1^ Department of Radiology, Jefferson Integrated Magnetic Resonance Imaging Center Thomas Jefferson University Philadelphia Pennsylvania USA; ^2^ Department of Neurosurgery Jefferson Integrated Magnetic Resonance Imaging Center Philadelphia Pennsylvania USA; ^3^ Department of Radiology Thomas Jefferson University Philadelphia Pennsylvania USA; ^4^ Department of Physical Therapy, Jefferson College of Rehabilitation Sciences Thomas Jefferson University Philadelphia Pennsylvania USA; ^5^ NeuroPoly Lab, Institute of Biomedical Engineering Polytechnique Montreal Montreal Quebec Canada

**Keywords:** MTR, normative data, pediatrics, spinal cord

## Abstract

**Background and Purpose:**

This study presents automated atlas‐based magnetization transfer (MT) measurements of the typically developing pediatric cervical spinal cord (SC). We report normative MT ratio (MTR) values from the whole cervical cord white matter (WM) and WM tracts, examining variations with age, sex, height, and weight.

**Methods:**

MT scans of 33 healthy females (mean age = 12.8) and 22 males (mean age = 13.09) were acquired from the cervical SC (C2–C7) using a 3.0 T MRI. Data were processed using the SC Toolbox, segmented, and registered to the PAM50 template. Affine and non‐rigid transformations co‐registered the PAM50 WM atlas to subject‐specific space. MTRs were measured for the specific WM tracts (left and right dorsal fasciculus gracilis, dorsal fasciculus cuneatus, and lateral corticospinal tracts [LCST]) and the whole WM. Descriptive statistics, correlation analysis, and unpaired *t*‐tests (*p* < 0.05) assessed relationships with age, height, weight, and sex.

**Results:**

Normative MTR measurements were obtained from all regions. The coefficients of variation were low to moderate. No significant differences (*p* > 0.05) were found across all the cervical levels. However, significant sex differences were observed in whole WM (*p* = 0.04) and LCST (*p* = 0.03). MTR values correlated positively with age, with significant correlations at C5 (*r* = 0.3, *p* false discovery rate = 0.04). A decreasing trend in MTR values across levels was found for whole WM (*r* = −0.2, *p* < 0.001).

**Conclusions:**

This study provides an understanding of MTR values in pediatric cervical SC and their variations by sex, age, height, and weight, providing a baseline for comparisons in pediatric SC diseases.

## 1 Introduction

Magnetization transfer (MT) imaging is a well‐established neuroimaging technique that has been used to detect tissue microstructural damage, specifically white matter (WM) tract integrity [1]. MT contrast relies on the interaction of hydrogen protons associated with macromolecules (such as lipids found in the myelin sheets), providing an indirect measurement of myelin content [2, 3]. This technique has been widely applied in demyelination disorders such as multiple sclerosis, cervical spondylotic myelopathy, and SC injury not only to detect changes in myelin but also to track disease progression and treatment effects [4, 5]. Furthermore, MT measurements have contributed to understanding the pathophysiology of neurological disorders [6, 7]. The application of this method is not limited to adults. MT has shown promising results in the pediatric population with neurological disorders such as medulloblastoma, hypoxic‐ischemic brain injuries, and epilepsy, all focusing on the brain [8, 9, 10]. In spinal cord (SC) imaging, particularly for adults, there are established normative MT values for whole cord and tract‐based evaluations, demonstrating the variability of the results based on age, sex, SC length, weight, and height [11]. However, limited work has been done to investigate normative values in pediatrics.

Despite the progress of MT imaging (MTI), there are obstacles to applying this technique in a clinical field, due to the broad range of normative values limiting its reliability [11]. Providing accurate normative values is especially important for pediatric population, due to the complex process of SC development, triggering particular conversions in its microstructural properties relevant to age [12].

Prior research has demonstrated the utility of MTR values in elucidating the myelination process across various brain ROIs throughout pediatric development [5, 13]. However, to the best of our knowledge, there are no studies investigating tract‐based or cross‐sectional MT ratio (MTR) changes of SC across various age groups of pediatrics, highlighting the necessity for establishing such values within this demographic [5, 14, 15].

The current study aims to measure the MTR values across the cervical levels and distinct spinal tracts among children aged 6–17, and report the normative MTR values for the pediatric population. This research also evaluates the correlation of these measures with age and sex, aiming to investigate any differences in pediatric SC maturation phases [16].

## 2 Methods

### 2.1 Study Design and Subjects

Fifty‐five typically developing (TD) children including a group of 33 females (mean age, 12.84 ± 2.8 years) and 22 males (mean age, 13.90 ± 2.1 years) were included in data analysis. This study was a cross‐sectional study conducted as part of a larger project, which involved recruiting children. Institutional review board approved informed consent from the child's legal guardian and written informed assent from the children, were obtained. Children included in this study had no past medical history of SC injuries, pathologies, or any neurological medical diagnosis, all being assessed by performing a screening questionnaire and a brief evaluation of their motor function.

### 2.2 MR Imaging Acquisitions

This study was conducted on a 3 Tesla Prisma MR scanner (Siemens Healthineers, Erlangen, Germany). Neck elements (4‐channel) of the 20‐channel head/neck coil and 24‐channel spine matrix coil were used. The imaging procedure followed established methods previously employed by one of the authors in the adult population (J.C.‐A.), ensuring consistency and comparability with previous studies. MTI was performed using a fast low‐angle shot sequence acquired in the axial plane parallel to the SC with parameters: field of view of 230 mm, acquisition matrix of 256 × 256, voxel size of 0.9 × 0.9 × 5 mm^3^, repetition time (TR) of 35 ms, echo time (TE) of 3.1 ms and 22 slices. The total MTI time was 4.24 min. Structural three‐dimensional (3D) T1‐weighted (W) and T2‐W scans were also performed for each subject for anatomic localization and template registration. 3D T2 (sampling perfection with application optimized contrast) images were acquired in the sagittal plane with parameters: field of view of 256 mm, acquisition matrix 320 × 320, slice thickness of 0.8 mm, 64 slices per slab, voxel size of 0.8 × 0.8 × 0.8 mm^3^, TR of 1500 ms, TE of 120 ms.

### 2.3 Image‐Analysis Techniques

MR images were analyzed using the SC Toolbox (V6.2, Montreal, Canada, https://spinalcordtoolbox.com/stable/index.html) [5, 6]. The pipeline used in this study for MT analysis and SC segmentation has been established and described elsewhere [11, 15, 17]. In brief, initially, MT data were first segmented using the structural T2‐W. To ensure high accuracy in segmentation, fiducials/landmarks were utilized. Specifically, landmarks were placed at the C2 and C5 levels manually to aid in automated segmentation. When necessary, manual segmentation was also employed after quality control of the segmentations, overseen by board‐certified neuroradiologist, with over 30 years of experience. This approach addressed and corrected any inconsistencies, optimizing the overall quality of the segmentations [6]. Next, the data were registered to the PAM50 template to align and spatially normalize individual data into a standard space for group comparison [15].

**FIGURE 1:**
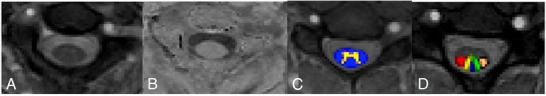
(A) MT1 (acquired with MT saturation pulses) image, (B) MTR image, (C) white matter/gray matter segmentation on T2‐weighted images, the blue area demonstrates white matter, and the yellow part shows the gray matter, (D) white matter atlas overlaid on the MT image. The red area on the right shows the right lateral corticospinal tract, the yellow, blue, and green areas represent dorsal columns, and on the left, the bright pink shows the left lateral corticospinal tract.

A sequence of flexible deformations was utilized to estimate affine transformations between the MT image and the template. These collective transformations allowed for the alignment of the PAM50 WM atlas to the unique anatomical space of each subject [15]. MTR values were extracted from specific WM tracts within the region of interest in the cervical cord, including the left and right dorsal fasciculus gracilis, dorsal fasciculus cuneatus, and lateral corticospinal tracts (LCST) tract (Figure 1).

### 2.4 Statistical Analysis

To assess the normative MTR values, the average and standard deviation (SD) of the MTR values were calculated according to sex, age, and vertebral level in each SC tract and the entire WM. An unpaired *t*‐test was performed for each tract to compare MTR values between the male and female participants. The differences in the MTR values across rostro caudal levels were evaluated with analysis of variance (ANOVA) for each tract and the whole WM. Subsequently, Spearman coefficients were employed to evaluate statistically significant differences between the mean MTR values and the vertebral levels, aiming to identify any associations. In addition, the coefficient of variation (CV) was computed using descriptive statistics to directly compare the variability of MTR values between different WM tracts using this formula:

$$CV=\left(\sigma /\mu \right)\times 100$$

where *σ* is the population SD and *μ* is the population mean MTR values. The CV for each tract was classified as low, if it was less than 5%, moderate if it was between 5% and 10%, and high if it was greater than 10% [18].

Finally, Spearman correlation analysis was performed for each tract and the whole WM to investigate the relationship between age, height, and weight with MTRs, and level‐based analysis was performed for the whole WM, motor and sensory tracts separately. All statistical analysis was performed using Prism GraphPad (V.9.4.1, GraphPad software, LLC, Boston, MA, USA, https://www.graphpad.com/features) and SPSS (V.29, IBM SPSS software, Armonk, New York, USA, https://www.ibm.com/spss).

## 3 Results

### 3.1 Tract‐Based MTR Normative Values

**FIGURE 2:**
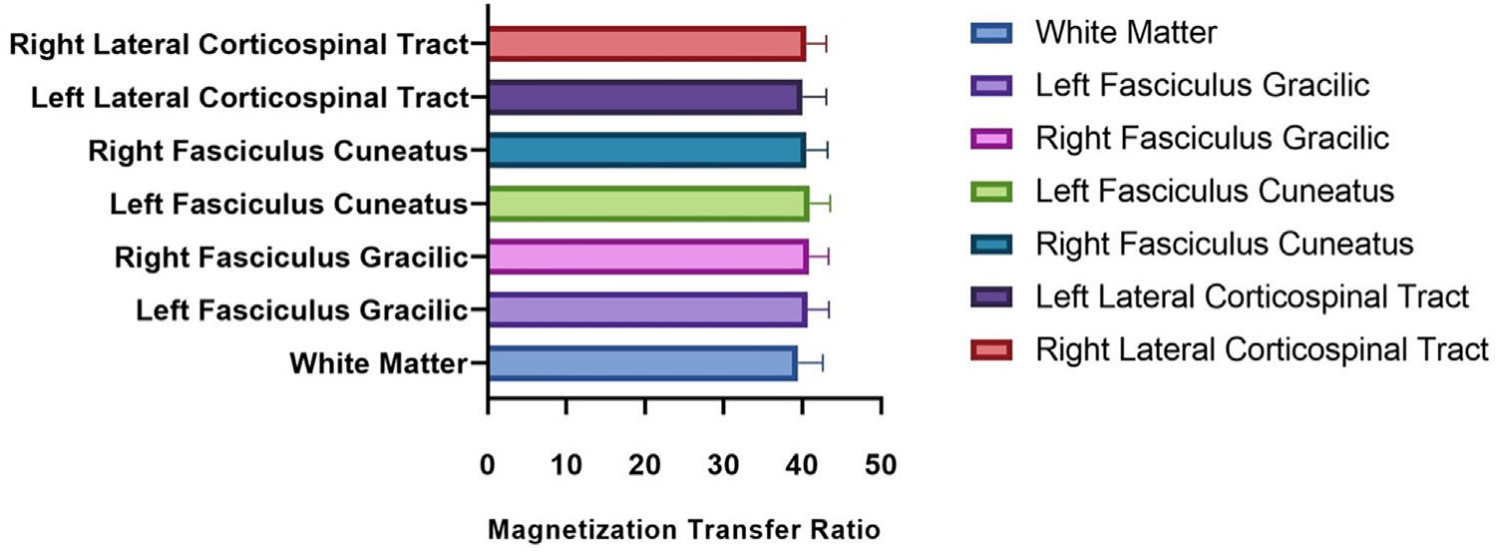
Magnetization transfer ratio normative values average across the entire cervical spinal cord white matter (WM) and three selected bilateral WM tracts. No significant differences were found across regions (*p* > 0.05).

MTR values were extracted from C2 to C7, separately for each SC tract. Figure 2 demonstrates the distribution of average MTR values in the SC tracts across subjects. The MTR CV for each tract showed moderate intersubject variability for most tracts and low variability for LCST. Table 1 shows the normative MTR values for different WM regions along with the CV measures.

**TABLE 1:** Mean of magnetization transfer ratio values across different white matter spinal cord regions and their coefficient of variation.

Spinal cord regions	Mean MTR ± standard deviation	Coefficient of variation (%)	90% Confidence interval
White matter	40.06 ± 2.5	6.2	2.3%, 15.1%
Left fasciculus gracilis	39.79 ± 2.7	6.8	3.1%, 21%
Right fasciculus gracilis	40.81 ± 2.5	6.1	2.8%, 22.6%
Left fasciculus cuneatus	40.95 ± 2.6	6.3	2.4%, 23%
Right fasciculus cuneatus	40.53 ± 2.6	6.4	3.2%, 18%
Left lateral corticospinal	40.02 ± 1.6	4	2.7%, 17.3%
Right lateral corticospinal	40.51 ± 2.5	6.1	3.4%, 20.6%

### 3.2 MTR Values by Rostro Caudal Level

**FIGURE 3:**
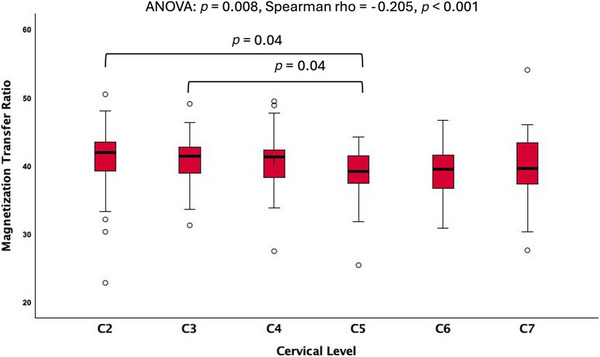
Magnetization transfer ratio (MTR) versus vertebral level for the whole cervical white matter. Showing significantly different values of MTR across levels (*p* < 0.05). ANOVA, analysis of variance.

ANOVA analysis found significant differences (*p* = 0.008) in MTR values across different rostro–caudal levels of the whole WM in the cervical SC, particularly between the C2/C3, and C5 levels (*p* = 0.04) (Figure 3). However, the differences in the MTR values from rostral to caudal cervical spine for distinct WM tracts were not significantly different (*p* > 0.05). Spearman coefficient analysis of the whole WM showed decreasing MTR values from rostral to caudal vertebral levels (Spearman's rho = −0.205, *p* < 0.001) (Figure 3).

### 3.3 MTR Variation with Demographics

**FIGURE 4:**
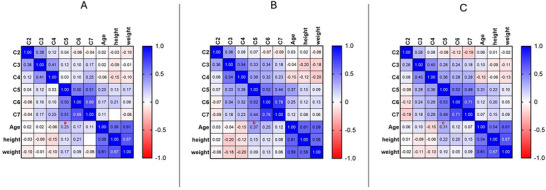
Correlation matrix of the whole white matter (WM) (A) motor tracts (B) and sensory tract's (C) magnetization transfer ratio (MTR) values with age, height and weight at each vertebral level. After applying false discovery rate (FDR) correction, the only significant correlation was found between age and MTR of the whole WM at C5 level representing as “a” in red (*p*‐FDR = 0.04), however, C5 showed consistent maximum correlation with age in all three regions of interest (a, b, and c).

MTR values were statistically different between females and males, with females showing higher MTR values in the CST by 37% (*p* = 0.03, *t* = 2.15) and in the entire WM by 33% (*p* = 0.04, *t* = 2.04) compared to males. No significant age differences were found between groups (*p *> 0.05). Correlation analysis showed an increasing however not statistically significant pattern of MTR values with age in all studied tracts (Table 2). Level‐based analysis showed an increasing trend of MTR values with age, with a significant positive correlation at C5 for the whole WM (*p* false discovery rate = 0.04, *r* = 0.3) (Figure 4). Height and weight showed no significant correlation with the MTR values.

**TABLE 2:** Relationship of the magnetization transfer ratio values across different white matter spinal cord tracts and age.

TRACTS	Age
Left fasciculus gracilis	*r* = 0.18, *p* = 0.1
Right fasciculus gracilis	*r* = 0.16, *p* = 0.2
Left fasciculus cuneatus	*r* = 0.03, *p* = 0.8
Right fasciculus cuneatus	*r* = 0.10, *p* = 0.4
Left lateral corticospinal	*r* = 0.22, *p* = 0.1
Right lateral corticospinal	*r* = 0.11, *p* = 0.3

## 4 Discussion

In this study, we measured the normative MTR values in the cervical SC of 55 TD children from various WM tracts and evaluating age and sex‐related differences. To the best of our knowledge, this study is the first to report the normative MTR values and demonstrate age and sex‐related differences of WM tracts in the cervical SC of the pediatric population. Previous studies have examined normative tract‐based diffusion imaging analysis metrics, such as fractional anisotropy (FA) and mean diffusivity (MD), which are well‐known diffusion tensor imaging (DTI) metrics used to explore the WM of the CNS in both adults and pediatric populations [19, 20]. In pediatrics, they reported an increase in FA and a decrease in MD with age throughout the SC. These changes are likely due to maturation processes, including an increase in axonal membranes and myelin density, leading to larger fiber tract diameters and resulting in increased FA. In addition, the decrease in MD is associated with a reduction in extracellular space and water content, reflecting myelination as the SC matures [17]. Furthermore, imaging metrics such as MT have been employed to assess the maturation of WM tracts in the brain, revealing decreasing values with age in adult populations, with significantly lower values after the third decade of life, along with notable differences between hemispheres and tract‐based diffusion analysis outcomes in the brain [21, 22]. However, in the pediatric population, MTR measures have shown a positive correlation between age and WM tracts of the brain, like the DTI metrics, peaking at 6 years old [22, 23]. Similar investigations on the SC revealed a declining trend in MT values with age in adults, with a mean age of 47.1 ± 15.3 [11]. Our findings showed an increasing trend in MTR values in the cervical SC with age, which aligns with previous reports and may reflect ongoing myelination processes in the pediatric population. Previous studies indicated that myelination occurs in the first 2–3 years of life and continues until early adulthood, peaking in the third decade of life before declining [24, 25].

We also performed level‐based correlation analysis with age, height, and weight for the whole WM, motor, and sensory tracts, showing a positive trend from C5 to C7, which was maximally correlated with age at the C5 level. This may be attributed to the C5 level being more susceptible to changes during the development of the cervical spine, potentially making it a critical area for detecting age‐related variations in younger individuals. Similar results revealed a negative correlation between the cross‐sectional area of the cervical SC and age in adults, particularly at the C5/C6 intervertebral level [26]. In addition, we investigated differences in MTR values between males and females, revealing significantly higher values in the whole WM and CST for females. Previous studies evaluating sex differences in the myelination process and cross‐sectional evaluation of the SC suggested that variations in spine or brain size and the regional distributions of gray matter (GM) and WM between males and females are predominantly associated with disparities in WM volume [27–29, 30]. Tract‐based DTI analysis of the brain in adolescents has shown significantly lower MD and higher FA in the CST bilaterally in females, reflecting greater WM density in the brain CST compared to males, which is consistent with our results in the tract‐based SC analysis [31, 32].

The concept of age‐ and sex‐related dissimilarities might be helpful to understanding the central nervous system's developmental processes and is essential for distinguishing between normal and atypical variations [33, 34]. Moreover, clarifying the connection between MTR and age in children's SC lays the foundation for creating more accurate standard databases similar to MTR normative values in adults, which have already has proven to be helpful in detecting SC pathologies [35, 36, 37].

To establish tract‐based normative MTR values, accurate segmentation of SC is a key step [38]. In this study, we successfully segmented the WM and GM, followed by the segmentation of WM tracts, as shown in Figure 1. The low to moderate CV values across different tracts demonstrate moderate intersubject variability presented in Table 1, which is consistent with previous studies, demonstrating segmentation approaches in SC imaging for adults [39, 40, 41]. These normative values could serve as a valuable reference for future studies, providing a foundation for assessing SC pathologies [42, 43, 44, 45].

To investigate the effects of anatomical variability across the SC in each vertebral level, we performed descriptive statistical MTR measures across the cervical vertebral levels for each tract. Prior studies have indicated that different vertebral levels exhibit unique structural characteristics that might impact the MTR metrics [46, 47]. Our findings, depicted in Figure 3, illustrate a decline in rostral–caudal MTR patterns within the cervical spine's WM. However, when evaluating tracts across vertebral levels, a linear pattern emerged, with no significant differences observed in MTR values.

Previous studies investigating the distribution of diffusion metrics along the entire SC in adults have reported a peak in FA and MT values at the C2 and C7 levels, along with a monotonic variation showing decreasing FA and MTR values and increasing MD values from rostral (C2) to caudal levels (C7). These findings are consistent with our observations in the pediatric population [11, 17]. The volume of WM progressively diminishes toward the caudal direction due to the reduced presence of axons within the long ascending and descending pathways as they traverse more caudal segments of the SC [48]. Furthermore, in the upper cervical cord, both the right‐to‐left diameter of the SC and the area of the central GM, are smaller compared to the lower cervical cord [49]. The observed rostro–caudal decrease in MTR values in this study may be attributed to a combination of factors, including changes in axonal density, myelination, WM volume, and structural differences along the rostro–caudal axis.

The small size of the SC in pediatric subjects makes the segmentation of the WM challenging and potentially could have contributed to the higher intersubject variability. The limited age range and small sample size may also have amplified these challenges. Future studies with larger, multicenter cohorts and the use of advanced machine learning techniques for segmentation could help overcome these limitations.

In conclusion, this study offers a comprehensive examination of SC MT properties, encompassing segmentation, normative values, sex differences, and age‐related changes in the pediatric population. These findings contribute to our understanding of SC microstructure and its variations across pediatric population. The results might have implications for future clinical practice, highlighting the importance of considering gender and age in SC evaluations.

## Conflicts of Interest

The authors declare no conflicts of interest.

## References

[1] A. R. Martin , B. De Leener , J. Cohen‐Adad , et al., “A Novel MRI Biomarker of Spinal Cord White Matter Injury: T2*‐Weighted White Matter to Gray Matter Signal Intensity Ratio,” American Journal of Neuroradiology 38 (2017): 1266–1273. 28428212 10.3174/ajnr.A5162 PMC7960075

[2] W. Kucharczyk , P. M. Macdonald , G. J. Stanisz , and R. M. Henkelman , “Relaxivity and Magnetization Transfer of White Matter Lipids at MR Imaging: Importance of Cerebrosides and pH,” Radiology 192, no. 2 (1994): 521–529. 8029426 10.1148/radiology.192.2.8029426

[3] G. B. Pike , N. De Stefano , S. Narayanan , et al., “Multiple Sclerosis: Magnetization Transfer MR Imaging of White Matter Before Lesion Appearance on T2‐Weighted Images,” Radiology 215, no. 3 (2000): 824–830. 10831705 10.1148/radiology.215.3.r00jn02824

[4] J. Cohen‐Adad , M. M. El Mendili , S. Lehéricy , et al., “Demyelination and Degeneration in the Injured Human Spinal Cord Detected With Diffusion and Magnetization Transfer MRI,” NeuroImage 55, no. 3 (2011): 1024–1033. 21232610 10.1016/j.neuroimage.2010.11.089

[5] B. De Leener , V. S. Fonov , D. L. Collins , V. Callot , N. Stikov , and J. Cohen‐Adad , “PAM50: Unbiased Multimodal Template of the Brainstem and Spinal Cord Aligned with the ICBM152 Space,” NeuroImage 165 (2018): 170–179. 29061527 10.1016/j.neuroimage.2017.10.041

[6] B. De Leener , S. Lévy , S. M. Dupont , et al., “SCT: Spinal Cord Toolbox, an Open‐Source Software for Processing Spinal Cord MRI Data,” NeuroImage 145 (2017): 24–43. 27720818 10.1016/j.neuroimage.2016.10.009

[7] A. R. Martin , I. Aleksanderek , J. Cohen‐Adad , et al., “Translating State‐of‐the‐Art Spinal Cord MRI Techniques to Clinical Use: A Systematic Review of Clinical Studies Utilizing DTI, MT, MWF, MRS, and fMRI,” NeuroImage: Clinical 10 (2015): 192–238. 26862478 10.1016/j.nicl.2015.11.019 PMC4708075

[8] R. Della Nave , A. Magaudda , R. Michelucci , et al., “Whole‐Brain Histogram and Voxel‐Based Analyses of Apparent Diffusion Coefficient and Magnetization Transfer Ratio in Celiac Disease, Epilepsy, and Cerebral Calcifications Syndrome,” American Journal of Neuroradiology 28, no. 3 (2007): 479–485. 17353316 PMC7977847

[9] J. H. Harreld , P. Zou , N. D. Sabin , et al., “Pretreatment Normal WM Magnetization Transfer Ratio Predicts Risk of Radiation Necrosis in Patients With Medulloblastoma,” American Journal of Neuroradiology 43, no. 2 (2022): 299–303. 35058296 10.3174/ajnr.A7393 PMC8985672

[10] P. Jissendi Tchofo , C. Christophe , P. David , T. Metens , G. Soto Ares , and D. Balériaux , “Apparent Diffusion Coefficient (ADC) and Magnetization Transfer Ratio (MTR) in Pediatric Hypoxic‐Ischemic Brain Injury,” Journal of Neuroradiology 32, no. 1 (2005): 10–19. 15798608 10.1016/s0150-9861(05)83016-0

[11] A. R. Martin , B. De Leener , J. Cohen‐Adad , et al., “Clinically Feasible Microstructural MRI to Quantify Cervical Spinal Cord Tissue Injury Using DTI, MT, and T2*‐Weighted Imaging: Assessment of Normative Data and Reliability,” American Journal of Neuroradiology 38, no. 6 (2017): 1257–1265. 28428213 10.3174/ajnr.A5163 PMC7960088

[12] I. S. Buyanova and M. Arsalidou , “Cerebral White Matter Myelination and Relations to Age, Gender, and Cognition: A Selective Review,” Frontiers in Human Neuroscience 15 (2021): 662031. 34295229 10.3389/fnhum.2021.662031 PMC8290169

[13] V. Xydis , L. Astrakas , A. Zikou , K. Pantou , S. Andronikou , and M. I. Argyropoulou , “Magnetization Transfer Ratio in the Brain of Preterm Subjects: Age‐Related Changes During the First 2 Years of Life,” European Radiology 16 (2006): 215–220. 15965662 10.1007/s00330-005-2796-8

[14] M. B. Cloney , Z. A. Smith , K. A. Weber II , and T. B. Parrish , “Quantitative Magnetization Transfer MRI Measurements of the Anterior Spinal Cord Region Are Associated With Clinical Outcomes in Cervical Spondylotic Myelopathy,” Spine 43, no. 10 (2018): 675–680. 29068880 10.1097/BRS.0000000000002470 PMC6621550

[15] S. Shahrampour , B. De Leener , M. Alizadeh , et al., “Atlas‐Based Quantification of DTI Measures in a Typically Developing Pediatric Spinal Cord,” American Journal of Neuroradiology 42, no. 9 (2021): 1727–1734. 34326104 10.3174/ajnr.A7221 PMC8423036

[16] M. Moccia , S. Ruggieri , A. Ianniello , A. Toosy , C. Pozzilli , and O. Ciccarelli , “Advances in Spinal Cord Imaging in Multiple Sclerosis,” Therapeutic Advances in Neurological Disorders 12 (2019): 1756286419840593. 31040881 10.1177/1756286419840593 PMC6477770

[17] S. Saksena , D. M. Middleton , L. Krisa , et al., “Diffusion Tensor Imaging of the Normal Cervical and Thoracic Pediatric Spinal Cord,” American Journal of Neuroradiology 37, no. 11 (2016): 2150–2157. 27418470 10.3174/ajnr.A4883 PMC7963763

[18] T. K. Koo and M. Y. Li , “A Guideline of Selecting and Reporting Intraclass Correlation Coefficients for Reliability Research,” Journal of Chiropractic Medicine 15, no. 2 (2017): 155–163. 10.1016/j.jcm.2016.02.012 PMC4913118 27330520

[19] M. Alizadeh , J. Fisher , S. Saksena , et al., “Age Related Diffusion and Tractography Changes in Typically Developing Pediatric Cervical and Thoracic Spinal Cord,” NeuroImage: Clinical 18 (2018): 784–792. 29876264 10.1016/j.nicl.2018.03.014 PMC5988463

[20] T. Y. Chan , X. Li , K. C. Mak , J. P. Cheung , K. D. Luk , and Y. Hu , “Normal Values of Cervical Spinal Cord Diffusion Tensor in Young and Middle‐Aged Healthy Chinese,” European Spine Journal 24 (2015): 2991–2998. 26208941 10.1007/s00586-015-4144-2

[21] N. C. Silver , G. J. Barker , D. G. MacManus , P. S. Tofts , and D. H. Miller , “Magnetisation Transfer Ratio of Normal Brain White Matter: A Normative Database Spanning Four Decades of Life,” Journal of Neurology, Neurosurgery, and Psychiatry 62, no. 3 (1997): 223–228. 9069474 10.1136/jnnp.62.3.223 PMC1064148

[22] C. Lebel , M. Gee , R. Camicioli , M. Wieler , W. Martin , and C. Beaulieu , “Diffusion Tensor Imaging of White Matter Tract Evolution Over the Lifespan,” NeuroImage 60, no. 1 (2012): 340–352. 22178809 10.1016/j.neuroimage.2011.11.094

[23] S. Uda , M. Matsui , C. Tanaka , et al., “Normal Development of Human Brain White Matter from Infancy to Early Adulthood: A Diffusion Tensor Imaging Study,” Developmental Neuroscience 37, no. 2 (2015): 182–194. 25791575 10.1159/000373885

[24] C. Lebel and S. Deoni , “The Development of Brain White Matter Microstructure,” NeuroImage 182 (2018): 207–218. 29305910 10.1016/j.neuroimage.2017.12.097 PMC6030512

[25] J. D. Yeatman , B. A. Wandell , and A. A. Mezer , “Lifespan Maturation and Degeneration of Human Brain White Matter,” Nature Communications 5, no. 1 (2014): 4932. 10.1038/ncomms5932 PMC4238904 25230200

[26] F. Kato , Y. Yukawa , K. Suda , M. Yamagata , and T. Ueta , “Normal Morphology, Age‐Related Changes and Abnormal Findings of the Cervical Spine. Part II: Magnetic Resonance Imaging of over 1,200 Asymptomatic Subjects,” European Spine Journal 21 (2012): 1499–1507. 22302162 10.1007/s00586-012-2176-4 PMC3535246

[27] T. J. Passe , P. Rajagopalan , L. A. Tupler , C. E. Byrum , J. R. MacFall , and K. R. Krishnan , “Age and Sex Effects on Brain Morphology,” Progress in Neuro‐Psychopharmacology & Biological Psychiatry 21, no. 8 (1997): 1231–1237. 9460088 10.1016/s0278-5846(97)00160-7

[28] J. S. Allen , H. Damasio , T. J. Grabowski , J. Bruss , and W. Zhang , “Sexual Dimorphism and Asymmetries in the Gray‐White Composition of the Human Cerebrum,” NeuroImage 18, no. 4 (2003): 880–894. 12725764 10.1016/s1053-8119(03)00034-x

[29] V. J. Schmithorst , S. K. Holland , and B. J. Dardzinski , “Developmental Differences in White Matter Architecture Between Boys and Girls,” Human Brain Mapping 29, no. 6 (2008): 696–710. 17598163 10.1002/hbm.20431 PMC2396458

[30] N. Papinutto , C. Cordano , C. Asteggiano , et al., “MRI Measurement of Upper Cervical Spinal Cord Cross‐Sectional Area in Children,” Journal of Neuroimaging 30, no. 5 (2020): 598–602. 32639671 10.1111/jon.12758 PMC7530010

[31] S. Bava , V. Boucquey , D. Goldenberg , et al., “Sex Differences in Adolescent White Matter Architecture,” Brain Research 1375 (2011): 41–48. 21172320 10.1016/j.brainres.2010.12.051 PMC3035918

[32] M. M. Silveri , M. L. Rohan , P. J. Pimentel , S. A. Gruber , I. M. Rosso , and D. A. Yurgelun‐Todd , “Sex Differences in the Relationship Between White Matter Microstructure and Impulsivity in Adolescents,” Magnetic Resonance Imaging 24, no. 7 (2006): 833–841. 16916700 10.1016/j.mri.2006.03.012

[33] A. Alizadeh , S. M. Dyck , and S. Karimi‐Abdolrezaee , “Traumatic Spinal Cord Injury: An Overview of Pathophysiology, Models and Acute Injury Mechanisms,” Frontiers in Neurology 10 (2019): 282. 30967837 10.3389/fneur.2019.00282 PMC6439316

[34] A. Cadotte , D. W. Cadotte , M. Livne , et al., “Spinal Cord Segmentation by One Dimensional Normalized Template Matching: A Novel, Quantitative Technique to Analyze Advanced Magnetic Resonance Imaging Data,” PLoS One 10, no. 10 (2015): e0139323. 26445367 10.1371/journal.pone.0139323 PMC4596853

[35] C. Malattia , M. Tolend , M. Mazzoni , et al., “Current Status of MR Imaging of Juvenile Idiopathic Arthritis,” Best Practice & Research Clinical Rheumatology 34, no. 6 (2020): 101629. 33281052 10.1016/j.berh.2020.101629

[36] F. Russo , L. Ambrosio , E. Giannarelli , et al., “Innovative Quantitative Magnetic Resonance Tools to Detect Early Intervertebral Disc Degeneration Changes: A Systematic Review,” Spine Journal 23, no. 10 (2023): 1435–1450. 10.1016/j.spinee.2023.05.011 37247638

[37] M. Seif , C. A. Gandini Wheeler‐Kingshott , J. Cohen‐Adad , A. E. Flanders , and P. Freund , “Guidelines for the Conduct of Clinical Trials in Spinal Cord Injury: Neuroimaging Biomarkers,” Spinal Cord 57, no. 9 (2019): 717–728. 31267015 10.1038/s41393-019-0309-x PMC6760553

[38] H. Rasoanandrianina , S. Demortière , A. Trabelsi , et al., “Sensitivity of the Inhomogeneous Magnetization Transfer Imaging Technique to Spinal Cord Damage in Multiple Sclerosis,” American Journal of Neuroradiology 41, no. 5 (2020): 929–937. 32414903 10.3174/ajnr.A6554 PMC7228162

[39] S. M. Dupont , B. De Leener , M. Taso , et al., “Fully‐Integrated Framework for the Segmentation and Registration of the Spinal Cord White and Gray Matter,” NeuroImage 150 (2017): 358–372. 27663988 10.1016/j.neuroimage.2016.09.026

[40] B. De Leener , M. Taso , J. Cohen‐Adad , and V. Callot , “Segmentation of the Human Spinal Cord,” Magma 29 (2016): 125–153. 26724926 10.1007/s10334-015-0507-2

[41] F. Prados , J. Ashburner , C. Blaiotta , et al., “Spinal Cord Grey Matter Segmentation Challenge,” NeuroImage 152 (2017): 312–329. 28286318 10.1016/j.neuroimage.2017.03.010 PMC5440179

[42] C. Granziera , J. Wuerfel , F. Barkhof , et al., “Quantitative Magnetic Resonance Imaging Towards Clinical Application in Multiple Sclerosis,” Brain 144, no. 5 (2021): 1296–1311. 33970206 10.1093/brain/awab029 PMC8219362

[43] B. Combès , A. Kerbrat , J. C. Ferré , et al., “Focal and Diffuse Cervical Spinal Cord Damage in Patients With Early Relapsing‐Remitting MS: A Multicentre Magnetisation Transfer Ratio Study,” Multiple Sclerosis 25, no. 8 (2019): 1113–1123. 29909771 10.1177/1352458518781999

[44] Y. Chen , E. M. Haacke , and E. Bernitsas , “Imaging of the Spinal Cord in Multiple Sclerosis: Past, Present, Future,” Brain Sciences 10, no. 11 (2020): 857. 33202821 10.3390/brainsci10110857 PMC7696997

[45] M. M. El Mendili , G. Querin , P. Bede , and P. F. Pradat , “Spinal Cord Imaging in Amyotrophic Lateral Sclerosis: Historical Concepts‐Novel Techniques,” Frontiers in Neurology 10 (2019): 350. 31031688 10.3389/fneur.2019.00350 PMC6474186

[46] J. L. Sherman , P. Y. Nassaux , and C. M. Citrin , “Measurements of the Normal Cervical Spinal Cord on MR Imaging,” American Journal of Neuroradiology 11, no. 2 (1990): 369–372. 2107721 PMC8334714

[47] H. Y. Ko , J. H. Park , Y. B. Shin , and S. Y. Baek , “Gross Quantitative Measurements of Spinal Cord Segments in Human,” Spinal Cord 42, no. 1 (2004): 35–40. 14713942 10.1038/sj.sc.3101538

[48] W. W. Campbell , DeJong's The Neurologic Examination. (Lippincott Williams and Wilkins, 2005).

[49] G. J. Barker , “Diffusion‐Weighted Imaging of the Spinal Cord and Optic Nerve,” Journal of the Neurological Sciences 186 (2001): S45–S49. 11334989 10.1016/s0022-510x(01)00490-7

